# Cerebral correlates of faking: evidence from a brief implicit association test on doping attitudes

**DOI:** 10.3389/fnbeh.2015.00139

**Published:** 2015-05-29

**Authors:** Sebastian Schindler, Wanja Wolff, Johanna M. Kissler, Ralf Brand

**Affiliations:** ^1^Affective Neuropsychology, Department of Psychology, University of BielefeldBielefeld, Germany; ^2^Center of Excellence Cognitive Interaction Technology, University of BielefeldBielefeld, Germany; ^3^Division of Sport and Exercise Psychology, University of PotsdamPotsdam, Germany

**Keywords:** EEG/ERP, implicit association test (IAT), faking, deception, indirect tests, anti-doping, right inferior frontal gyrus

## Abstract

Direct assessment of attitudes toward socially sensitive topics can be affected by deception attempts. Reaction-time based indirect measures, such as the Implicit Association Test (IAT), are less susceptible to such biases. Neuroscientific evidence shows that deception can evoke characteristic ERP differences. However, the cerebral processes involved in faking an IAT are still unknown. We randomly assigned 20 university students (15 females, 24.65 ± 3.50 years of age) to a counterbalanced repeated-measurements design, requesting them to complete a Brief-IAT (BIAT) on attitudes toward doping without deception instruction, and with the instruction to fake positive and negative doping attitudes. Cerebral activity during BIAT completion was assessed using high-density EEG. Event-related potentials during faking revealed enhanced frontal and reduced occipital negativity, starting around 150 ms after stimulus presentation. Further, a decrease in the P300 and LPP components was observed. Source analyses showed enhanced activity in the right inferior frontal gyrus between 150 and 200 ms during faking, thought to reflect the suppression of automatic responses. Further, more activity was found for faking in the bilateral middle occipital gyri and the bilateral temporoparietal junction. Results indicate that faking reaction-time based tests alter brain processes from early stages of processing and reveal the cortical sources of the effects. Analyzing the EEG helps to uncover response patterns in indirect attitude tests and broadens our understanding of the neural processes involved in such faking. This knowledge might be useful for uncovering faking in socially sensitive contexts, where attitudes are likely to be concealed.

## Introduction

Attitudes are among the strongest social cognitive predictors of human behavior (Kraus, 1995). Direct (i.e., self-report) assessment of socially sensitive attitudes can be distorted by social desirability bias (McDaniel et al., 2009) because the purpose of a given test often is easy to determine, and thus allows participants to deliberately choose and alter their responses (Roehner et al., 2011). The Implicit Association Test (IAT; Greenwald et al., 1998) constitutes a class of reaction-time based indirect tests that aim to hide the true goal of measurement better than do direct tests. It is presented typically as a lexical sorting task on a computer, where two concepts (one target and one evaluative) are mapped on the same response key of the keyboard. The task is easier and reaction times are faster when the two concepts that share the same response key (e.g., flowers + like) are closely associated, rather than when the they are not associated (e.g., insects + like).

IAT methods have evolved as one standard for indirect attitude testing in social cognition research (Krosnick et al., 2005). One of the IAT's most important features is its postulated potential to control for the social desirability bias by evading voluntary control and being rather robust toward deception attempts compared to direct tests (Kaempfe et al., 2009; Teige-Mocigemba et al., 2010). Indeed, compared with questionnaires, IATs display higher predictive validity when socially sensitive constructs are measured (Greenwald et al., 2009). As a more economic, but equally valid and reliable variant, Brief IATs (BIAT) have received considerable scientific attention in the past few years (Sriram and Greenwald, 2009).

Doping attitudes are among the strongest statistical predictors of doping behavior (e.g., Mallia et al., 2013; Ntoumanis et al., 2014). Doping in sports is a socially and legally sanctioned behavior. Therefore, people with rather permissive doping attitudes are often motivated to disguise their real attitude and instead provide the socially desired response, namely that they dislike doping (Gucciardi et al., 2010). Whereas, data from doping attitude questionnaires is often skewed and of very limited value for the prediction of doping behavior, the doping BIAT (Brand et al., 2014a) has been found to be a valid predictor for positive biochemical doping test results (Brand et al., 2014b). Thus, in the present study, the behavioral and neural correlates of faking the doping BIAT are examined.

In general, the IAT's robustness toward faking has been heavily studied as of late (Fiedler and Bluemke, 2005; De Houwer et al., 2007; Cvencek et al., 2010; Roehner et al., 2013). We use the terms faking and deception synonymously in this article because the former is more common in the cited social cognition research (e.g., Fiedler and Bluemke, 2005; De Houwer et al., 2007), whereas the latter is more common in the neuroscientific research we cite (e.g., Johnson et al., 2003, 2004, 2005, 2008; Crites et al., 2010). So far, results have indicated that the IAT can be deceived to some extent (e.g., Fiedler and Bluemke, 2005; De Houwer et al., 2007). However, most participants need to be instructed regarding a successful faking strategy. Kim (2003), for example, showed that participants could not effectively conceal their positive attitude toward flowers unless they were told *how* to do so. Only after having been provided with the explicit strategy to respond more slowly when the concepts *flower* and *like* were mapped on the same response key did participants not reveal their positive attitude. Recently, considerable research efforts also have been devoted to autobiographical IAT (aIAT) faking. Here, the truthfulness of a previously established autobiographic memory is evaluated using reaction-time based IAT methodology (for a review, see Agosta and Sartori, 2013). Regarding faking the aIAT, response slowing likewise has been demonstrated as an effective means to fake this test (Verschuere et al., 2009). In addition, a recent study (Hu et al., 2012) has demonstrated the possibility to fake the aIAT by speeding up responses in the incongruent block. However, the aIAT differs from traditional IAT variants in that it focuses on autobiographical memories (Hu et al., 2012) and not on social cognitive predictors of behavior, such as attitudes (Greenwald et al., 1998).

There are theoretical and practical reasons for why research on IAT faking has become topical in the social cognition literature: Evidence showing that IATs can be faked has challenged the theoretical claim that IAT scores really reflect implicit associations. These are theorized to represent output from the impulsive system of the social information system (De Houwer et al., 2009) and should therefore be immune to faking. From a practical perspective, test-takers with high motivation to disguise their true attitude will most likely begin to develop and apply deception strategies. It is thus important to investigate possibilities to detect fake test results (e.g., Cvencek et al., 2010) and potential threats to test validity in general.

Extant studies only address overt behavioral consequences of deception attempts on IAT variants (i.e., changes in reaction times) or try to statistically detect faking (e.g., Agosta et al., 2010; Cvencek et al., 2010), but do not consider cerebral mechanisms. Whereas, no research has yet addressed the involved cerebral correlates of IAT deception, a few studies have addressed the cerebral processes involved in completing an IAT in general (Ibáñez et al., 2010; Williams and Themanson, 2011; Forbes et al., 2012). For instance, Forbes et al. (2012) found a large, early positivity over frontal and occipital regions, and tighter synchronization between these regions, specifically for blocks where attitude and response key were congruent. The authors interpreted this synchronization as reflecting a close match between brain regions involved in sensory processing and those involved in executive functions. This, in turn, was interpreted as support for the notion that the IAT actually measures automatic associations.

Faking and deception, in general, have been studied using EEG, most often investigating guilty-knowledge paradigms. Overall, results have suggested that there is no *specific lie response* in ERPs (Johnson et al., 2008). Rather, ERP differences may strongly reflect the involved cognitive processes. For example, Hu and Rosenfeld (2012) investigated groups of participants who were either instructed to commit a mock crime or not. When presenting “guilty” participants with rare crime-relevant—compared to frequent crime-irrelevant—stimuli, these participants showed an increased P300 compared to an “innocent” control group that was shown rare information that was autobiographical, but not related to the mock crime. This is in line with increased P300 amplitudes often found in oddball experiments for novel stimuli, and for stimuli that are inconsistent with the established context or inconsistent with participants' attitudes (Cacioppo et al., 1993; Ito and Urland, 2003, 2005; Dickter and Bartholow, 2007). However, Hu et al. (2011) also found a *decrease* in P300 for deceptive responses in a design where participants had to make an equal number of honest and deceptive responses. The same pattern of results was also found for the later occurring LPP. Crites et al. (2010) found an increased LPP when participants misreported attitudes toward rare pictures or names, but a *decreased* LPP when comparing deceptive to truthful responses toward frequent pictures or names (Crites et al., 2010). Thus, a decrease in the P300 and LPP over central locations is frequently reported when participants give an equal number of deceptive responses to previously learned stimuli (Johnson et al., 2003, 2004, 2005). Regarding deceitful reporting of personal attitudes, participants who were instructed to lie about their own previously assessed attitudes also showed a decreased P300/LPP over fronto-central sites, whereas they showed an increased positivity over occipital regions (Johnson et al., 2008). It might be that the visual processing of the faking stimuli seems to have been down-regulated, suggesting perceptual disengagement from critical target stimuli as one mechanism of successful faking. Previous research has indicated that P300 amplitudes decrease as the cognitive resources needed by a secondary task increase (Johnson, 1986). Thus, in balanced faking designs, a decrease in the P300/LPP is related to the amount of monitoring processes needed and cognitive control required (Johnson et al., 2008).

More recently, even earlier differences are reported when participants faked responses to self-related vs. non-self-related information. Previously for the N1 and N2, an increased negativity was found for faking (Hu et al., 2011). This could reflect the conflict between the automatic and the response actually given, as an increased N2 is also found for responses to incongruent prime-target pairs (Bartholow et al., 2009). In line with this, increased N1 and N2 were also found when participants had to inhibit responses in NOGO tasks compared to equally frequent GO tasks. These differences were linked to enhanced activity in right inferior frontal regions (Lavric et al., 2004).

In previous deception studies, participants responded either truthfully or deceitfully to stimuli in yes/no forced-choice formats. We aimed to apply these findings to reaction-time based tests. We therefore investigated the cerebral correlates of faking an attitude test by enabling test takers to alter their responses in a test where faking is difficult (i.e., when participants are not informed of how this test can be faked), but whose socially sensitive content induces participants to do so (e.g., see Wolff et al., 2015). In line with the experimental paradigm most often used in social cognition research on IAT faking, participants were given an explicit faking strategy (see Kim, 2003; Fiedler and Bluemke, 2005; Cvencek et al., 2010; Roehner et al., 2013). Response slowing on one's true attitude is the most commonly implemented strategy (Verschuere et al., 2009.), although it also seems possible to cheat on at least some variants of the IAT via response acceleration (Hu et al., 2012). As response slowing has been used more often in the literature, and as response acceleration suffers from the problem that there is a natural lower limit on reaction times—such that if participants really show full effort on baseline testing, they may not be able to go much faster—response slowing was the faking instruction chosen in the present study.

Participants were required to either respond honestly or to try faking the doping BIAT using the strategy provided (BIAT; Brand et al., 2014a). The field of doping attitude testing promises high ecological validity, with our task being a realistic simulation of what is likely to happen when athletes undergo respective psychometric testing. This can serve as a baseline against which the doping BIAT results obtained from athletes can be compared. The full sequence of early ERPs was investigated to determine the onset of faking instruction effects. Using a balanced design containing an equal number of truthful and deceptive responses, it was hypothesized that fake responses on the BIAT, similar to deceptive responses in other contexts, should lead to an increased occipital positivity (Johnson et al., 2008) and an increased frontal N1 and N2 (Hu et al., 2011; Hypothesis 1).

Further, a decrease of the P300/LPP over central sites, which is consistently found for deceptive responses in various experiments and is interpreted to reflect increased task demands (Johnson et al., 2003, 2004, 2005, 2008; Crites et al., 2010; Hu et al., 2011), should be present when participants fake reactions to the BIAT (Hypothesis 2). Finally, whereas the scalp topography of the observed differences give rough cues about their possible cortical origin, EEG source estimation using inverse modeling can reveal the likely generators more precisely. Specifically, right prefrontal regions have been implicated in the inhibition of pre-potent motor responses (Garavan et al., 1999; Bellgrove et al., 2004; Lavric et al., 2004; Nee et al., 2007; Ye and Zhou, 2009). Consequently, enhanced activity in right prefrontal regions is predicted for faking blocks in which an automatic response has to be inhibited and slowed (Hypothesis 3).

## Methods

### Participants

Twenty-four students were recruited at the University of Bielefeld. They gave written informed consent and received course credit for participation. The study was conducted in accordance to the Declaration of Helsinki and was approved by the ethics review board at the University of Bielefeld. One participant was excluded due to a history of previous mental disorder, another due to a previous brain tumor, and two participants due to excessive artifacts, leaving 20 participants for final analysis. One participant was left-handed.

These 20 participants (15 females) were 24.65 years of age, on average (*SD* = 3.50, *Min* = 20, *Max* = 30). Screenings with the German version of the Beck Depression Inventory and the State Trait Anxiety Inventory (Spielberger et al., 1999; Hautzinger et al., 2009) revealed neither clinically relevant depression (*M* = 4.25, *SD* = 3.46) nor anxiety scores (*M* = 30.00; *SD* = 3.60).

### Design

We used a counterbalanced within-group (repeated measures) design to test how faking a positive doping attitude (*faking positive, 40 trials*), faking a negative doping attitude (*faking negative, 40 trials*) or a veridical test (*baseline, 40 trials*) affected BIAT scores and EEG signals. As IAT faking has been found to be virtually impossible when participants complete the test for the first time (Fiedler and Bluemke, 2005), all participants completed one practice BIAT first. Then, participants worked on a sequence of three BIATs. Prior to completing each BIAT, participants received either the standard instruction (*baseline*) or were instructed how to fake a positive (*faking positive*) or a negative (*faking negative*) doping attitude. Counterbalancing of sequences (instructions) resulted in six conditions that participants were randomly assigned to (see Table 1).

**Table 1:** **Research design**.

***t***	**Counterbalanced sequence of experimental BIAT instructions**						***Block* discrimination**	***Trials***
1	Practice	Practice	Practice	Practice	Practice	Practice	Doping + Like^a^	20
							Doping + Dislike	20
2	Faking negative	Faking negative	Faking positive	Faking positive	Baseline	Baseline	Doping + Like	40
							Doping + Dislike	40
3	Faking Positive	Baseline	Baseline	Faking Negative	Faking Positive	Faking Negative	Doping + Like	40
							Doping + Dislike	40
4	Baseline	Faking Positive	Faking Negative	Baseline	Faking Negative	Faking Positive	Doping + Like	40
							Doping + Dislike	40

a *Whether doping + like or doping + dislike was presented as the first block was counterbalanced in order to avoid order effects*.

### The BIAT

Doping attitudes were assessed using a validated picture-based doping BIAT (Brand et al., 2014a). Our picture-based doping BIAT used the standard BIAT setup (Sriram and Greenwald, 2009). It required the combined classification of the two concept categories *doping* vs. *health food*, with the classification of the two attribute categories *like* vs. *dislike*. The doping BIAT's consists of two combined task blocks. In Block A, stimuli that belong to the concept *doping* or the attribute *like* must be categorized using the “I” key. In block B, *doping* stimuli and stimuli belonging to the attribute category *dislike* are mapped on the same response key, and must be categorized using the “I” key. As *doping* is consistently mapped on the “I” key, it is the *focal* concept because participants have to primarily attend to it (Sriram and Greenwald, 2009). Upon starting either combined task block, the complete stimulus set of the categories are shown on two introductory screens to allow for participants' familiarization with the stimuli (*doping* + *like* on one, *doping* + *dislike* on the next screen.) The stimuli of the non-focal category *health food* were not shown. The task-relevant category labels (*doping* + *like*, or *doping* + *dislike*) remain visible at the top and bottom of the screen so that participants know at any time what stimuli are focal and have to be categorized using the “I” key. The picture stimuli representing each category were selected based on an evaluation of their associative strength with their respective reference category (Brand et al., 2014a). The *doping* concept was represented by pictures of pills, ampoules, and syringes; the *health food* concept by apples, cereal, and vegetables; the *like* attribute by positive emoticons; and the *dislike* attribute by negative emoticons. According to the notation of Sriram and Greenwald, this setup corresponds to a doping–dislike/like–(health food) BIAT (2009). The BIAT program file and all stimuli used are made fully available in Brand et al. (2014a).

Inquisit 3.0 software (www.millisecond.com) was used to program the BIAT. The practice BIAT consisted of a discrimination block (20 trials) where participants were familiarized with the BIAT procedure. Then, the social expectations compatible block (*doping* + *dislike*, 20 trials) was presented, followed by the incompatible block (*doping* + *like*, 20 trials). The order of compatible and incompatible blocks was counterbalanced between participants to avoid positioning effects. In the following + trials, the discrimination block was removed, and compatible and incompatible blocks were expanded to 40 trials each. Our + is therefore identical to the one described by Brand et al. (2014a), with the exception that (a) we expanded to 40 trials to get an adequate number of trials per cell for ERP averaging, and (b) set the inter-trial interval to 1000 ms in order to avoid introducing artifacts into the EEG measure. D-scores are calculated according to the D4 algorithm such that negative scores represent a negative attitude toward doping (Greenwald et al., 2003). In the D4 algorithm, reaction times above 10,000 ms, and those of error trials, are deleted and are replaced by an error value (average reaction time of this participant in all correct trials of the block plus 600 ms; mere elimination of error trials would have a negative impact on the reliability of the test).

### BIAT faking instruction

In the faking negative condition, participants were instructed to fake the subsequent BIAT in a way that would seem like their attitude was strongly toward anti-doping. In line with previous deception research, participants were provided with an explicit faking strategy: For faking a positive attitude, participants were instructed to slow their responses when *doping* and *dislike* shared the same response key. In the faking negative condition, slowing of responses in the *doping* and *like* condition was described to be the faking strategy.

### EEG recording

EEG signals were recorded from 128 BioSemi active electrodes (www.biosemi.com) with a sampling rate of 2048 Hz. During recording, Cz was used as a reference electrode. Biosemi uses two separate electrodes as ground electrodes: First, a Common Mode Sense active electrode (CMS), and second, a Driven Right Leg passive electrode (DLR). Four additional electrodes (EOG) measured horizontal and vertical eye movement. These were placed at the outer canthi of the eyes and below the eyes.

Pre-processing and statistical analyses of source activity were done using SPM8 for EEG (http://www.fil.ion.ucl.ac.uk/spm/). Although perhaps best known as a toolbox for the analysis of functional magnetic resonance data, SPM provides a unitary framework for the analysis of neuroscience data acquired with different technologies, including EEG and MEG, using the same rationale (Penny and Henson, 2007; Litvak et al., 2011). In a first step, data were offline re-referenced to whole-scalp average reference. That is, for each measured time, the average voltage across all measured electrodes is subtracted from each electrode, resulting in non-zero voltage measurements for all 128 electrodes. To identify artifacts caused by saccades (horizontal, HEOG) or eye blinks (vertical, VEOG), virtual HEOG and VEOG channels were created from the EOG electrodes. EEG signals that were highly correlated with HEOG or VEOG activity were subtracted from the EEG (minimum correlation of 0.5). Data were then down-sampled to 250 Hz, and later band-pass filtered from 0.166 to 30 Hz with a fifth-order Butterworth zero-phase filter. Filtered data were segmented from 100 ms before stimulus onset until 1000 ms after stimulus presentation. 100 ms before stimulus onset were used for baseline correction. Automatic artifact detection was used to eliminate remaining artifacts defined as trials exceeding a threshold of 150 μV (see e.g., Küper et al., 2012; Cecchini et al., 2013; Kuipers and Thierry, 2013; Schindler et al., 2014). Data were then averaged using the robust averaging algorithm of SPM8, excluding possible further artifacts. Robust averaging down-weights outliers for each channel and each measured time, thereby preserving a higher number of trials. This is because artifacts are not supposed to distort the whole trial, but most of the time corrupt only parts of the trial. We used the recommended offset of the weighting function, which preserves approximately 95% of the data points drawn from a random Gaussian distribution (Litvak et al., 2011). Overall, 3.12% of all electrodes were interpolated, and 20.74% of all trials were rejected. From an initial 40 trials for each block (doping + like and doping + dislike) within the three conditions (baseline, faking negative, faking positive), we were able to retain 31.7 trials, on average. Conditions did not differ with regard to number of useable trials *F*_(2, 38_) = 0.98, *p* = 0.38, partial η^2^ = 0.05, and there was no interaction between block and condition *F*_(2, 38)_ = 0.61, *p* = 0.55, partial η^2^ = 0.03.

Source reconstructions of the cortical generators of significant ERP differences were calculated and statistically assessed with SPM8 for EEG (Friston et al., 2008; Lopez et al., 2013), following recommended procedures. First, a realistic boundary element head model (BEM) was derived from SPM's template head model based on the standard brain from the Montreal Neurological Institute (MNI brain). Electrode positions then were transformed to match the template head, which is thought to generate reasonable results even when individual subjects' head differ from the template (Litvak et al., 2011). Average electrode positions, as provided by BioSemi, were co-registered with the cortical mesh template for source reconstruction. Group inversion (Litvak and Friston, 2008) was computed and the multiple sparse priors algorithm implemented in SPM8 was applied. This method allows activated sources to vary in the degree of activity, but restricts the activated sources from being the same in all subjects (Litvak and Friston, 2008). This is thought to result in more precise source estimation than single-subject matrix inversion. For source reconstruction, frequency contents between 0.166 and 30 Hz were analyzed (Litvak et al., 2011). For each analyzed time window, three-dimensional source reconstructions were generated as NIFTI images. These images were smoothed using an 8 mm full-width half-maximum kernel (voxel size = 2 mm × 2 mm × 2 mm).

### BIAT analyses

In order to test whether faking instructions worked, a repeated-measures ANOVA (condition: baseline, faking negative, faking positive) was set-up to investigate main effects for the resulting *D*-scores. *D*-scores are already an aggregate measure of reaction time differences between the doping + like (positive sign) and the doping + dislike block (negative sign). For significant effects (*p* < 0.05), *post-hoc* comparisons were computed to investigate direction of differences. Effect sizes were calculated for all statistical tests (Cohen, 1988). For significant violations of Mauchly's Assumption of Sphericity, degrees of freedom were corrected according to Greenhouse-Geisser.

### EEG data analyses

EEG scalp data were analyzed with EMEGS (http://www.emegs.org/, Peyk et al., 2011). For statistical analyses, 2 (block: doping + like vs. doping + dislike) × 3 (condition: baseline, faking negative, faking positive) repeated measures ANOVAs were set-up to investigate interaction effects between block and condition in time windows and electrode clusters of interest. We expected interaction effects because the ERPs for both blocks (doping + like and doping + dislike) were thought to differ depending on the given baseline (full effort) or faking instruction (negative vs. positive). For the faking positive condition, faking was expected to alter responses in the doping + dislike block, whereas for faking negative condition, faking was expected to alter responses in the doping + like block. For significant interaction effects, *post-hoc* comparisons were computed between the two blocks to investigate the direction of mean differences.

**Figure 1:**
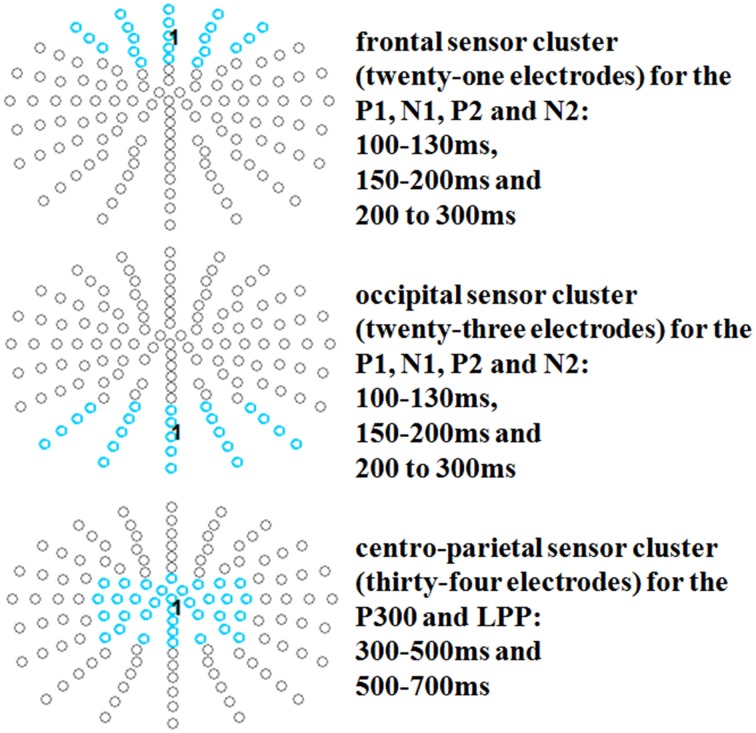
**Selected electrode clusters for all time windows**.

After identification of the ERP components, time windows were segmented from 100 to 130 to investigate occipital P1 and frontal N1 effects; from 150 to 200 ms to investigate occipital N1 and frontal P2 effects; from 200 to 300 ms to investigate occipital P2 and frontal N2 effects; and from 300 to 500 ms and 500 to 700 ms to investigate P3/LPP effects. For the early time window (P1-N2), an occipital cluster (twenty-three electrodes: PO5, PO7, PO9h, PO9, PO3, POO3, O1, OI1, I1, POz, POOz, Oz, OIz, Iz, PO4, POO4, O2, OI2, I2, PO6, PO8, PO10h, PO10; see e.g., Johnson et al., 2008), and also for the N1, P2/N2 time window, a frontal cluster was examined (twenty-one electrodes: AF7, AFF5, F3, Fp1, AFp3, AF3, AFF1, F1, Fpz, AFpz, AFz, AFFz, Fz, Fp2, AFp4, AF4, AFF2, F2, AF8, AFF6, F4; see e.g., Hu et al., 2011). For the P3/LPP time windows, a centro-parietal cluster was examined (thirty-four electrodes: FC3, C3, CP3, P3, FC3h, C3h, CP3h, P3h, FC1, C1, CP1, P1, FCC1h, CCP1h, FCz, Cz, CCPz, CPz, CPPz, Pz, FCC2h, CCp2h, FC2, C2, CP2, P2, FC4h, C4h, CP4h, P4h, FC4, C4, CP4, P4; see e.g., Johnson et al., 2005; Crites et al., 2010 and see Figure 1).

Statistical tests for source estimations were calculated for the same time windows as for the investigated scalp ERPs when significant scalp effects were found. In order to account for the noisier estimations in source space, the two faking blocks of both faking conditions were compared to the baseline blocks using a threshold of *p* < 0.005 (Campo et al., 2013; Schindler et al., 2015) with a minimum of 25 significant voxels (Schindler et al., 2015; Sun et al., 2015). The identification of involved brain regions was performed using the AAL atlas (Tzourio-Mazoyer et al., 2002).

## Results

### Effects of faking instruction on BIAT scores

**Figure 2:**
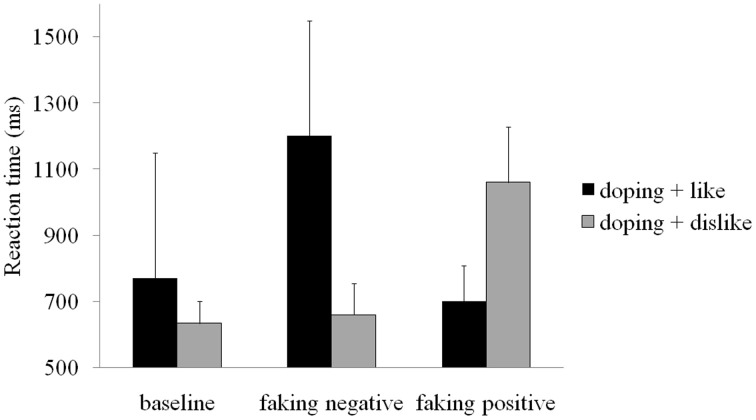
**Mean reaction times for each block and condition**. Error bars represent standard deviations.

Mean reaction times for each block in each condition are displayed in Figure 2. These raw reaction times show a slowing in the doping + dislike block for the faking positive condition and in the doping + like block in the faking negative condition. Participants' average doping attitudes in the baseline BIAT were somewhat negative (*D*-score = −0.26, *SD* = 0.54) and differed from zero, *t*_(19)_ = 2.14, *p* < 0.05, *d* = 0.98.

Repeated measures ANOVA showed a main effect of condition, *F*_(2, 38)_ = 123.00, *p* < 0.001, partial η^2^ = 0.87. *Post-hoc* comparisons showed that the D score in the faking positive condition (*D*-score = 1.27, *p* < 0.001, *d* = 2.43) was significantly larger than the baseline D-score (*D*-score = −0.26), which, in turn, was significantly larger than the D-score in the faking negative condition (*D*-score = −0.99, *p* < 0.001, *d* = −2.23). This indicates that participants were successful in behaviorally faking positive and negative doping attitudes.

### EEG results

**Figure 3:**
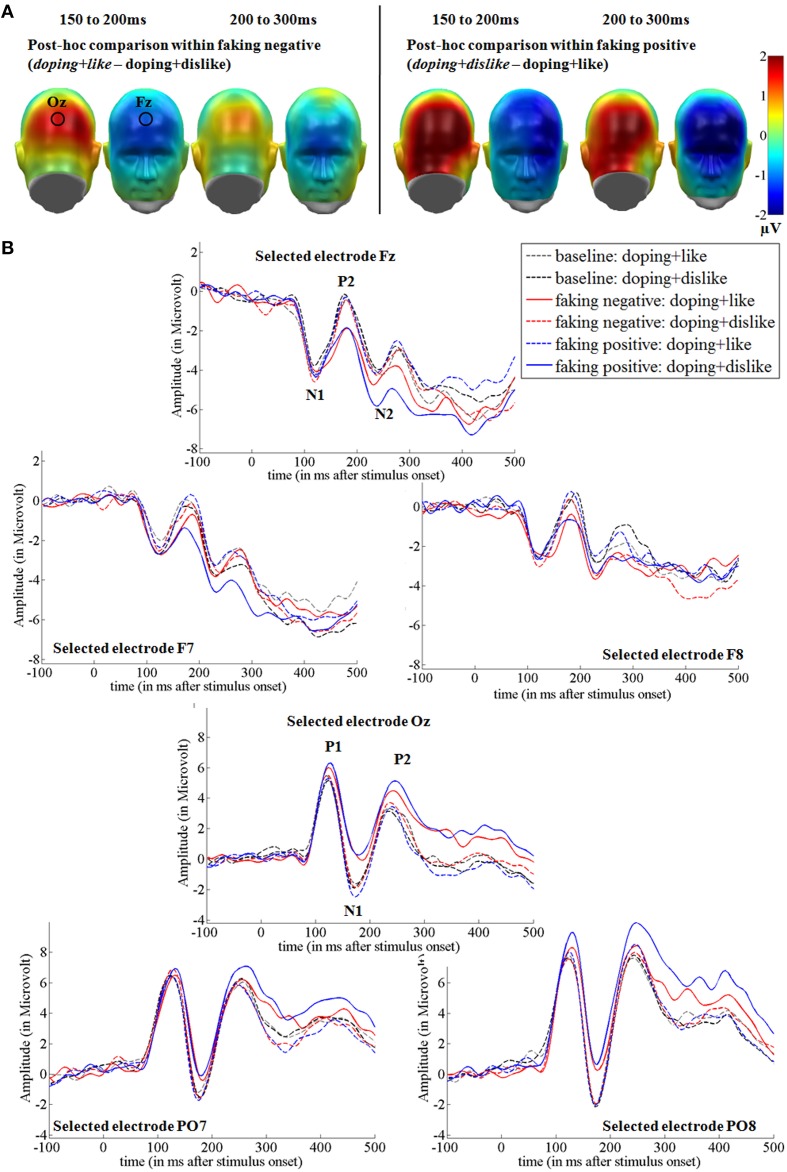
**Faking effects on the N1, P2, and N2 components. (A)** Difference topographies within the faking negative and the faking positive condition: Blue color indicates more negativity and red color indicates more positivity for the faking blocks (in italics). **(B)** Selected electrodes F7, Fz, and F8 for the frontal electrode cluster and PO7, Oz, and PO8 for the occipital electrode set, displaying the time course over frontal and occipital sites. Within the faking condition, the respective blocks where participants were instructed to delay their responses are represented by straight lines, whereas baseline blocks are illustrated by dotted lines.

**Figure 4:**
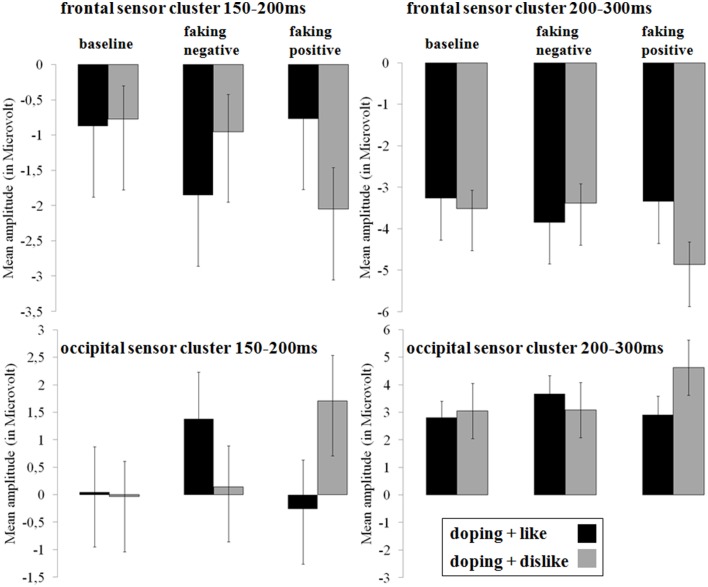
**Mean amplitudes in microvolt for early effects over frontal and occipital sensor clusters**. Error bars represent ±1 standard error of the mean (SEM).

**Figure 5:**
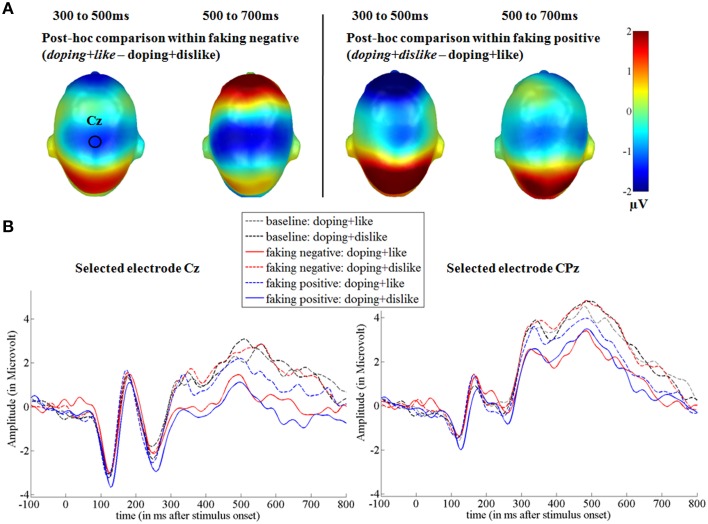
**Faking effects on the P300 and LPP components. (A)** Difference topographies within the faking negative and the faking positive condition: Blue color indicates more negativity and red color indicates more positivity for the faking blocks (in italics). **(B)** Selected electrodes Cz and CPz for the centro-parietal electrode cluster displaying the time course over central sites. Within the faking condition, the respective blocks where participants were instructed to delay their responses are represented by straight lines, whereas baseline blocks are illustrated by dotted lines.

**Figure 6:**
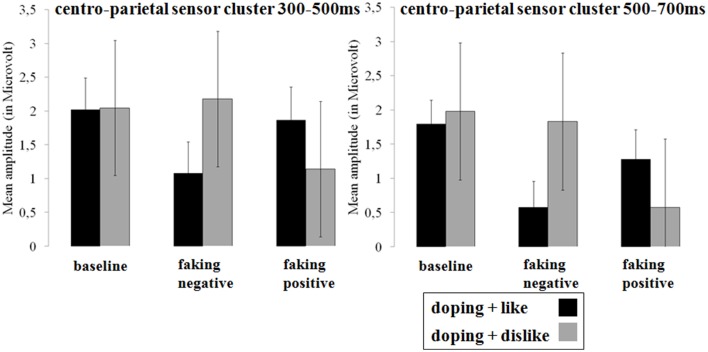
**Mean amplitudes in microvolt for late effects over the centro-parietal sensor cluster**. Error bars represent ±1 standard error of the mean (SEM).

In the two faking conditions, participants were instructed to either respond slower in blocks where like and doping shared the same key (faking negative), or where dislike and doping shared the same key (faking positive). Figures 3, **5** show the *post-hoc* comparisons within both faking conditions between the respective faking and baseline block. Figures 4, **6** show mean amplitudes in microvolt for all investigated time windows and sensor clusters. For comparisons between the two baseline blocks, no significant differences were found in any time window.

#### Occipital P1 and frontal N1 (100–130 ms). hypothesis 1 for early effects: increased frontal negativity and occipital positivity for faking

Between 100 and 130 ms, no significant interaction between block (block: doping + like vs. doping + dislike) and condition (condition: baseline, faking negative, faking positive) was found for the P1 component over occipital regions [*F*_(2, 38)_ = 1.92, *p* = 0.16, partial η^2^ = 0.09]. Further, between 100 and 130 ms, there was also no interaction for the frontal N1 [*F*_(2, 38)_ = 0.50, *p* = 0.61, partial η^2^ = 0.03].

#### Occipital N1 and frontal P2 (150–200 ms). hypothesis 1 for early effects: increased frontal negativity and occipital positivity for faking

For the occipital N1, a significant interaction was observed over occipital [*F*_(2, 38)_ = 18.32, *p* < 0.001, partial η^2^ = 0.49] regions between 150 and 200 ms. For faking, both the faking block of the faking negative condition (*p* < 0.01, *d* = 0.35) and of the faking positive condition (*p* < 0.001, *d* = 0.51) elicited a reduced negativity compared to the baseline blocks (see Figures 3, 4). By contrast, no differences were found between the baseline blocks (*p* = 0.79, *d* = 0.03).

Over frontal sites, a significant interaction was found at the P2 [*F*_(2, 38)_ = 13.76, *p* < 0.001, partial η^2^ = 0.42; see Figures 3, 4]. *Post-hoc* comparisons revealed that the faking block in the faking negative condition (*p* < 0.05, *d* = 0.35) and the faking block in the faking positive condition (*p* < 0.001, *d* = 0.50) were less positive-going compared to the baseline blocks in both faking conditions, leading to a decreased frontal P2. There was no difference between the two baseline blocks (*p* = 0.74, *d* = 0.05).

#### Occipital P2 and frontal N2 (200–300 ms). hypothesis 1 for early effects: increased frontal negativity and occipital positivity for faking

Between 200 and 300 ms, the interaction effects remained significant over occipital [*F*_(1.42, 27.06)_ = 9.41, *p* < 0.01, partial η^2^ = 0.33] and frontal regions [*F*_(1.53, 29.06)_ = 5.73, *p* < 0.05, partial η^2^ = 0.23]. For the occipital cluster, *post-hoc* comparisons showed that the faking block of the faking positive condition (*p* < 0.01, *d* = 0.58) elicited a larger positivity compared to the baseline block, leading to an increased P2, whereas no differences occurred between baseline blocks (*p* = 0.31, *d* = 0.10) and faking negative blocks (*p* = 0.15, *d* = 0.22).

Over frontal sites, the faking block of the faking positive condition led to an increased N2 compared to the baseline block (*p* < 0.01, *d* = 0.57), but there were neither significant differences between baseline blocks (*p* = 0.32, *d* = 0.13) nor between the two faking negative blocks (*p* = 0.31, *d* = 0.20).

#### P300 and LPP (300–700 ms). hypothesis 2 for late effects: decreased centro-parietal positivity for faking

Between 300 and 500 ms, a significant interaction was found over centro-parietal sites [*F*_(1.54, 29.32)_ = 6.84, *p* < 0.01, partial η^2^ = 0.27; see Figures 5, 6]. For both faking blocks of the faking negative condition (*p* < 0.01, *d* = 0.53) and of the faking positive condition (*p* < 0.05, *d* = 0.32), decreased P300 components were found compared to the baseline blocks. No significant differences were observed between baseline blocks (*p* = 0.90, *d* = 0.01).

In the last time window between 500 and 700 ms, a significant interaction was again observed over centro-parietal sites [*F*_(2, 38)_ = 11.26, *p* < 0.001, partial η^2^ = 0.37]. Again, compared to the baseline blocks, decreased amplitudes were found for both faking blocks of the faking negative condition (*p* < 0.001, *d* = 0.68) and of the faking positive condition (*p* < 0.05, *d* = 0.36), whereas no significant differences were found between baseline blocks (*p* = 0.38, *d* = 0.13).

### Source analyses. hypothesis 3: increased right-inferior frontal activity for faking

For time windows where differences in signal space had been found, source analyses were conducted to examine the differences in source activity between baseline and faking blocks. Table 2 provides detailed results.

**Table 2:** **Source analyses for each time window**.

**No. of sig. voxels per cluster**	**Peak *t*_(1, 78)_**	**Peak *p*(unc)**	**MNI space coordinates**			**AAL**
			***x* (mm)**	***y* (mm)**	***z* (mm)**	**Area label**
**FAKING > BASELINE 150–200 ms**						
431	4.18	<0.001	30	−88	6	Middle occipital R
215	3.93	<0.001	−54	−64	10	Middle temporal L
148	3.80	<0.001	50	−62	14	Middle temporal R
302	3.42	<0.001	−32	−78	10	Middle occipital L
105	3.30	<0.001	−64	−36	0	Middle temporal L
147	3.00	<0.005	42	40	−4	Inferior frontal R
**FAKING > BASELINE 200–300 ms**						
45	2.96	<0.005	50	−62	14	Middle temporal R
53	2.92	<0.005	−54	−62	8	Middle temporal L

*Comparisons are calculated between baseline and faking blocks (t-contrasts). No. of sig. voxels per cluster, number of significant voxels for each cluster. unc, uncorrected p-value. Each cluster may exhibit more than one peak, whereas only one peak is displayed. Peak coordinates are displayed in MNI space (x,y, and z). The identification of area labels for each peak was performed using the AAL atlas. R/L, right or left hemisphere*.

**Figure 7:**
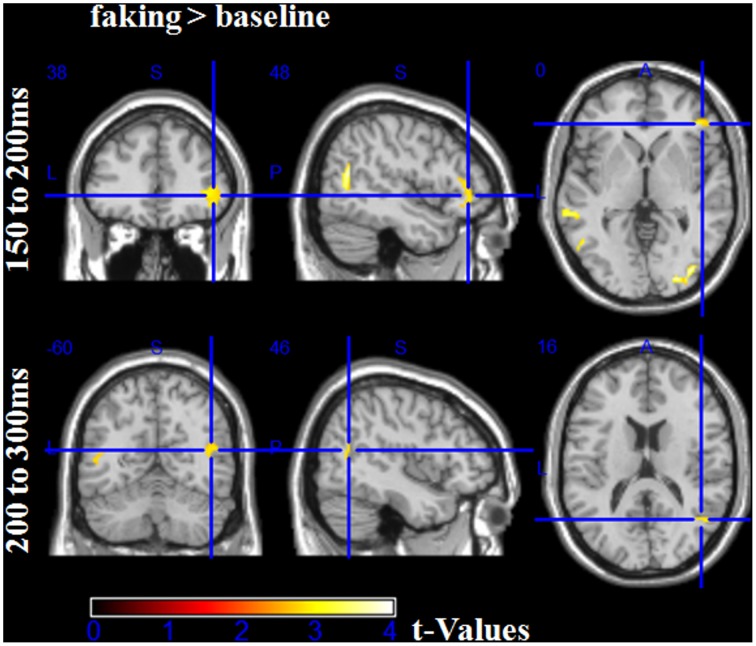
**Source estimations for the comparison between faking and baseline blocks for significant scalp effects**. In the first time window from 100 to 150 ms, more activity in faking blocks were observed in the right inferior frontal gyrus and bilaterally at the temporoparietal junction and middle occipital gyri. In the time window between 200 and 300 ms, more activity was observed bilaterally at the temporoparietal junction.

For the first time window between 150 and 200 ms, the faking blocks led to an enhanced activity in the right inferior frontal gyrus (largest peak [*t*_(1, 78)_ = 3.00, *p* < 0.005]), bilaterally in the middle occipital gyri (largest peak right [*t*_(1, 78)_ = 4.18, *p* < 0.001]), and bilaterally in the temporoparietal junction (TPJ, largest peak left [*t*_(1, 78)_ = 3.93, *p* < 0.001], see Figure 7). Importantly, there was no significantly large activity in source space for baseline blocks compared to faking blocks, even using an extremely liberal threshold (uncorrected *p* < 0.05).

Between 200 and 300 ms, in faking blocks, again, enhanced activity could be observed in the bilateral TPJ (largest peak [*t*_(1, 78)_ = 2.96, *p* < 0.005]). For the later time windows, no significant differences were found for any comparison, which may be partly explained by noisier estimations due to the longer time windows and by a potentially more complex generator structure.

## Discussion

We experimentally instructed participants to fake positive doping attitudes or fake negative doping attitudes, or to respond truthfully to a doping BIAT. Participants were given a faking strategy and thus could successfully fake doping attitude measurements in both directions. Descriptively, behavioral (IAT *D*-scores) and neuroscientific results (EEG data) suggest that faking a positive doping attitude was more effortful. This may be due to the already somewhat negative doping attitude participants exhibited in the baseline condition (*D*-score = −0.26).

Investigating the cerebral processing in all conditions, we found large ERP differences for faking. For the earliest investigated components—the occipital P1 and frontal N1—no faking effects were observed, suggesting no differences between the conditions in initial sensory processing. However, as expected, a more negative-going potential was observed for faking, starting at the P2. This negative-going frontal ERP for faking is in line with previous findings of a larger frontal N1 and N2 when lying (Hu et al., 2011). Whereas, Hu et al. (2011) investigated deceptive responses in a yes–no forced choice test, we examined reaction times on an attitude measure. In both cases (choosing the wrong answer or deliberately delaying responses), participants have to counteract their spontaneous, possibly automatic response tendency. Interestingly, larger N1 and N2 components can also be found when participants have to inhibit responses in NOGO tasks (Lavric et al., 2004) or are presented with incongruent targets, where primes prepared them for another response (Bartholow et al., 2009). Both the NOGO N2 and the conflict N2 has been subsumed to the control-related frontal N2 (Folstein and Van Petten, 2008). We infer from this that the increased frontal negativity could reflect the conflict between such an automatic response tendency and the deliberately given response. This frontal negativity seems to be influenced not only by the faking instruction, but also by the *direction* of the faking instruction. The presented stimuli and the faking strategy were identical for the faking negative and faking positive doping attitude conditions. Still, a larger N2 for faking was only observed within the faking positive condition, where the conflict between an automatic and a given response might have been more difficult to resolve. This could have been more demanding because it deviated more from the mildly negative doping attitudes of our participants.

Over occipital locations, a reduced negativity for the N1 and a larger positivity at the P2 was found while faking. An increased positivity over occipital sensors is also reported for deceptive responses toward previously assessed attitudes (Johnson et al., 2008). This reduced negativity at occipital sensors could reflect an alteration of visual information processing at secondary processing stages. Whereas, the P1 response was unaffected by the instruction, starting with the N1 visual processing of the target stimuli seems to have been down-regulated compared to baseline, suggesting perceptual disengagement from critical target stimuli as one mechanism of successful faking. Attention research often has shown increased N1 components for attended stimuli (Hillyard et al., 1998). These have been suggested to originate mainly in the secondary visual cortex (e.g., Hopf et al., 2002).

Later, starting with the P300 time window, less positive-going ERPs could be observed at central sites during both faking blocks. This is in accordance with the frequently reported decreased P300/LPP amplitudes for deceptive responses to previously shown stimuli or previously assessed attitudes (Johnson et al., 2003, 2004, 2008; Crites et al., 2010). In these studies, participants were instructed to choose the *wrong* response option while faking. Although there may be no *specific lie response* in ERPs (Johnson et al., 2008), current results suggest that there are striking similarities in the underlying processes when comparing these previous results with current findings from an experiment where participants were required to only alter their response speed.

Further, source analyses revealed that deception led to a larger activity in the right inferior frontal gyrus and, although not specifically hypothesized, bilaterally in the temporoparietal junction (TPJ) and the bilateral middle occipital gyri at earlier measured times. Previous research has shown that the right inferior frontal gyrus is important for the processing of attitudes (Wood et al., 2005; Johnson et al., 2011), but also for memory inhibition (Wimber et al., 2008) and inhibition of automatic responses (Garavan et al., 1999; Bellgrove et al., 2004; Nee et al., 2007; Ye and Zhou, 2009). Thus, the enhanced activity in faking blocks appears to reflect the inhibition of the automatic response to the presented stimulus. This inhibition might closely be related to response inhibition, for example, for memory inhibition, an increased late positivity over right frontal regions has been found (Hanslmayr et al., 2009). This response-related inhibition is further supported by similar source estimation results from an experiment using equally frequent GO and NOGO trials (Lavric et al., 2004). For NOGO trials where participants had to inhibit a response, larger source activity was found in right prefrontal areas in the N2 time window (220–320; Lavric et al., 2004). In this time window, response inhibition and conflict monitoring are discussed in the ERP literature (Lavric et al., 2004; Folstein and Van Petten, 2008; Bartholow et al., 2009). The combination of findings in scalp and source space therefore suggests an inhibitory account. We suppose that participants indeed had difficulties to overcome their pre-potent motor response when delaying their responses. Moreover, this initial right IFG activity may initialize the monitoring process that leads to reduced P3 and LPP components.

Also, while faking, participants showed more activity in the TPJ between 150 and 300 ms. Interestingly, this region has been previously shown to be more active when participants fake responses (Ganis et al., 2011). But as this region is also relevant for attention (Pessoa et al., 2009) and intentional actions (Saxe et al., 2004; den Ouden et al., 2005), its enhanced activity could also reflect the monitoring of the planned response, namely, to inhibit the automatic response toward the target stimulus.

Summarizing the results, subsequent deception can already be observed, starting with the frontal P2 and occipital N1, extending into the frontal N2 and occipital P2 and the centro-parietal P300/LPP. One could argue that the slowing of responses simply reduced ERP amplitudes throughout all time windows. However, the pattern of results in scalp and source space for the early components seem to be very similar to response inhibition findings (Lavric et al., 2004), whereas slower response behavior in the absence of inhibition requirements has been found to affect mostly the P300 (Wascher et al., 1996). In support of this argument, the frontal N2 and the occipital P2 are significantly larger in the critical condition of the *fake positive* block, which can be assumed to induce more of a response conflict and more inhibitory requirements than the *fake negative* block, which effectively only requires accentuation of the actual response tendency. By contrast, faking negative and faking positive does not differ on the parietal P3 and LPP components. Further, the enhanced source activity for the faking blocks in the TPJ and right inferior frontal gyrus suggests that even when a strategy is supplied, faking is likely an active cognitive process. The right inferior frontal gyrus activity may be responsible for suppressing an automatic response tendency.

So far, it is not clear whether the present results are specific to the BIAT or extend to other variants of the IAT, or in how far they also apply to slowing in reaction time-based tasks in general. ERP similarities between our results and those from other studies using forced-choice formats could support the hypothesis that our results are not BIAT specific. However, in light of frameworks that distinguish between automatic (implicit) and deliberate (explicit) attitudes (i.e., dual-process models of social cognition; Strack and Deutsch, 2004; Gawronski and Bodenhausen, 2006), our findings nevertheless suggest that conflicts between deliberate and automatic processes occur during faking, and that they may occur very early in the processing stream. Still, it is important to note that such early effects, as described by Hu et al. (2011) and in the present study, may be partly due to experimental design. The blocked designs used in these studies enable participants to anticipate and prepare deceptive responses across an entire block, which may have induced earlier ERP effects.

In sum, this is the first study to examine the neural correlates of faking the BIAT. It revealed that deception already modulates very early brain responses, and suggests the right inferior frontal gyrus to be a crucial brain region for suppressing automatic responses in the deception context. Further, source estimations suggest that the TPJ may be involved in the monitoring of executed responses and also of suppressed responses. Alternatively, these results show that IATs can be faked (in line with e.g., Kim, 2003), challenging the theoretical claim that IAT scores really reflect implicit associations. These are theorized to represent output from the impulsive system and should be immune to faking (De Houwer et al., 2009). From a practical perspective, test-takers with high motivation to disguise their true attitude will most likely begin to develop and apply deception strategies. Faking on socially sensitive topics (such as doping in sports) is therefore likely to occur for people who have a high intrinsic motivation to deceive on this topic (such as professional athletes). Knowledge of the cerebral processes that accompany deceptive efforts might be utilized in order to prevent or detect faking attempts in the future.

### Conflict of interest statement

The authors declare that the research was conducted in the absence of any commercial or financial relationships that could be construed as a potential conflict of interest.
