# Supplementary material for: Cerebral correlates of faking: evidence from a brief implicit association test on doping attitudes
Source: Front Behav Neurosci. 2015 May 29;9:139. doi: 10.3389/fnbeh.2015.00139 (PMC4448510; doi:10.3389/fnbeh.2015.00139)
Supplement: Supplementary file 1 [file DataSheet1.DOCX]

**Supplementary file 1: Additionally reported EEG main effects for all investigated time windows and sensor groups.**

**1. Occipital P1 and frontal N1: 100-130 ms**

Between 100 and 130ms, no main effect of condition (*F*_(2, 38)_ = 2.17, *p*=.13, partial η²=.10) and no main effect of block (*F*_(1, 38)_ = 3.75, *p*=.07, partial η²=.17) were found for the P1 component over occipital regions.

Further, between 100 and 130ms there also no main effect of condition (*F*_(2, 38)_ = 0.83, *p*=.45, partial η²=.04) and no main effect of block (*F*_(1, 38)_ = 1.98, *p*=.18, partial η²=.09) were found for the frontal N1.

**2. Occipital N1 and frontal P2: 150-200 ms**

For the occipital N1, a significant main effect of condition (*F*_(2, 38)_ = 5.62, *p*<.01, partial η²=.23) was observed. Here, the baseline condition was significantly more negative going compared to the faking conditions (*p*<.05). No difference was found between faking conditions (*p*=.88). No main effect of block (*F*_(1, 38)_ = 1.25, *p*=.28, partial η²=.06) was found for the occipital N1.

Over frontal sites a significant main effect of condition was found at the P2 (*F*_(2, 38)_ = 4.64, *p*<.05, partial η²=.20). Here, the baseline condition was significantly more positive going compared to the faking conditions (*p*<.05). No difference was found between faking conditions (*p*=.96). No main effect of block (*F*_(1, 38)_ = 0.27, *p*=.61, partial η²=.01) was found.

**3. Occipital P2 and frontal N2: 200-300 ms**

Between 200 and 300ms, the main effect of condition remained significant over occipital sensors (*F*_(2, 38)_ = 7.27, *p*<.01, partial η²=.28). Here, the baseline condition was significantly less positive going compared to the faking conditions (*p*<.05). Further, the faking positive condition was significantly more positive going compared to the faking negative condition (*p*<.05). No main effect of block (*F*_(1, 38)_ = 4.35, *p*=.05, partial η²=.19) was found.

Over frontal sites a main effect of condition was found at the N2 (*F*_(2, 38)_ = 6.81, *p*<.01, partial η²=.26). Here, the baseline condition was significantly less negative going compared to the faking positive condition (*p*<.01) but not different to the faking negative condition (*p*=.28). Further, the faking positive condition was significantly more negative going compared to the faking negative condition (*p*<.05). No main effect of block (*F*_(1, 38)_ = 3.99, *p*=.06, partial η²=.17) was found.

**4. P300 and LPP: 300-700ms**

Between 300 and 500ms, a significant main effect of condition was found over centro-parietal sites (*F*_(2, 38)_ = 4.42, *p*<.05, partial η²=.19). The baseline condition was significantly more positive going compared to the faking positive condition (*p*<.05) and as a trend compared to the faking negative condition (*p*=.07). No difference was found between faking conditions (*p*=.44). There was no main effect of block (*F*_(1, 38)_ = 1.77, *p*=.20, partial η²=.09).

In the last time window between 500 and 700ms, a significant main effect of condition was found over centro-parietal sites (*F*_(2, 38)_ = 15.26, *p*<.001, partial η²=.45). The baseline condition was significantly more positive going compared to the faking conditions (*p*<.01). The faking negative condition was also more positively going compared to the faking positive condition (*p*<.05). There was no main effect of block (*F*_(1, 38)_ = 4.18, *p*=.06, partial η²=.18).
